# Use of Anthropometric Data for the Prediction of Four-Strand Hamstring Graft Size in White Caucasian Population

**DOI:** 10.3390/jcm14030825

**Published:** 2025-01-27

**Authors:** Theodoros Bouras, Ioanna Lianou, Andreas Filippopoulos, John Lakoumentas, Dimitrios Ntourantonis

**Affiliations:** 1 Orthopedic Department, General Hospital of Patras, Kalavriton 37, 26332 Patras, Greece; theo_bouras@hotmail.com (T.B.); andreas.filippopoulos@gmail.com (A.F.); 2 Department of Medical Physics, School of Health Sciences, University of Patras, 26505 Rio, Greece; john.lakoo@gmail.com; 3 Emergency Department, University Hospital of Patras, 26504 Patras, Greece; d_douradonis@yahoo.gr

**Keywords:** ACL, hamstrings graft, diameter, length

## Abstract

**Background/Objectives:** The purpose of this study was to preoperatively estimate the four-strand hamstring graft size in a White Caucasian population, using anthropometric data. **Methods:** This was a prospective study of a consecutive series of fifty patients with anterior cruciate ligament (ACL) rupture, who were scheduled for reconstruction using hamstring autografts; however, one of them was ultimately not enrolled according to the exclusion criteria (49 patients in total). Preoperatively, age, sex, weight, body mass index (BMI), length, and diameter of the contralateral thigh, length of the harvested gracilis/semitendinosus tendons, and length and thickness of the four-stranded graft were recorded. Graft diameter and length were dependent variables, measured via a graft sizer and a ruler, respectively. Quantitative variables were described with mean ± SD (correlated in a pairwise manner with Pearson’s r correlation). Qualitative variables were described with an absolute count (relative % percent) per categorical level, and their dependency on any quantitative (dependent) variable was assessed via Student’s *t*-test. **Results:** The mean lengths of the gracilis and semitendinosus were 25.6 ± 3.2 cm and 28.4 ± 3.3 cm, respectively, and they were positively correlated with the length of the four-strand hamstring graft along with the patients’ height and thigh length. **Conclusions:** The use of anthropometric data can assist in the prediction of the hamstring autograft size, aiding the selection of an appropriate graft type. The four-strand hamstring graft length was related to the gracilis, semitendinosus, and thigh length. The patients’ height was related to the graft length and diameter.

## 1. Introduction

ACL injuries represent almost half of all knee injuries, being the most commonly injured ligament in the knee [1]. ACL rupture injury may be an outcome of several activities, i.e., pivoting sports or non-contact injuries. Ruptures from non-contact injuries tend to be an economically important disease, which could be explained by specific anatomic intra-articular structures or fatigue mechanisms [2]. Fatigue of the hamstring muscles can even impair the ability of the central nervous system to regain proprioceptive reweighting in case of postural changes [3]. Simultaneous damage to other knee tissues, including menisci, hyaline cartilage, or ligaments can cause degenerative changes, thus necessitating meticulous physical examination and imaging techniques for early diagnosis and treatment. According to the known literature, these techniques include clinical diagnostic tests of great sensitivity, accompanied by magnetic resonance imaging (MRI) [4,5]. Finally, these type of injuries seems to be widespread, especially within athletes of pivoting sports whose functional post-surgical outcomes seems to better upon returning to activity, particularly professional football players, comparing with those with non-contact ACL tears [6].

Despite continuous progress in ACL reconstruction, there are still many controversies about the most suitable graft [7]. Quadruple hamstring autograft, including semitendinosus and gracilis tendons, is the most frequently used graft in primary reconstruction by the majority of knee surgeons [1]. However, increased re-rupture rates compared to other autograft choices have been reported, stating that the size of the graft plays a huge role in avoiding failures [8,9]. Newer studies suggest that increases of even 0.5 mm up to a graft size of 10 mm can lead to better results, with the mean diameter of a four-strand hamstring graft ranging from 7.7 mm to 8.5 mm [9,10,11]. The preoperative prediction or estimation of the quadruple hamstring graft is an ongoing field of research. A plethora of studies over the last fifteen years have positively correlated anthropometric data with the four-strand graft diameter and length, reporting various correlations such as age, height, body mass index (BMI), gender, and thigh length and circumference [8,12,13,14,15,16,17,18,19,20,21]. More interestingly, other similar studies have focused on specific ethnic groups [22,23,24,25,26,27,28,29]. The primary aim of this study was to correlate the anthropometric parameters and patient characteristics of a White Caucasian population with the length and diameter of four-stranded hamstring grafts. The main aim was to establish the preoperative predictability (or lack thereof) of these anthropometric data.

## 2. Materials and Methods

### 2.1. Population Eligibility

This was a single-center prospective study, incorporating consecutive patients with ACL rupture who were scheduled for reconstruction between February 2023 and March 2024. The inclusion criteria included patients between 16 and 50 years old, both males and females with a recent injury (less than six months) in the lower limbs, without any previous knee injury of the ipsilateral limb. All participants needed to be able to give verbal consent and comply with the treatment, rehabilitation, and follow-up protocols. Patients younger than 16 and older than 50 years old were excluded. Moreover, the exclusion criteria included patients requiring multiligamentous reconstruction, patients with previous knee injuries or lower limb injuries treated with major surgical procedures, and patients requiring the use of an allograft for the reconstruction. The clinical or radiological presence of osteoarthritis or inflammatory or crystalline arthropathies also led to exclusion from this study. The study was conducted in accordance with the Declaration of Helsinki and approved by the Institutional Review Board (or Ethics Committee) of the General Hospital of Patras (protocol code 4449/24).

### 2.2. Surgical Procedure

**Figure 1:**
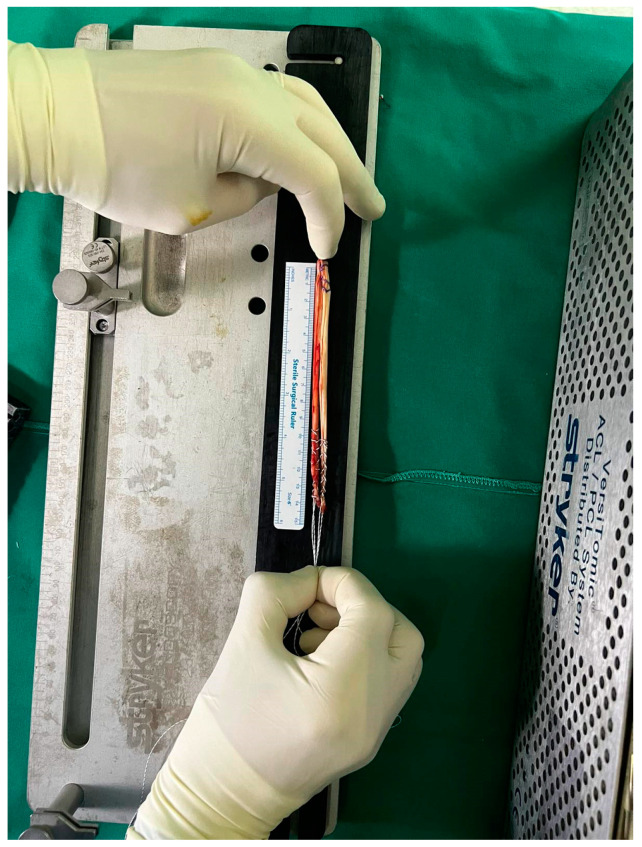
Length measurement of the four-strand hamstring graft.

**Figure 2:**
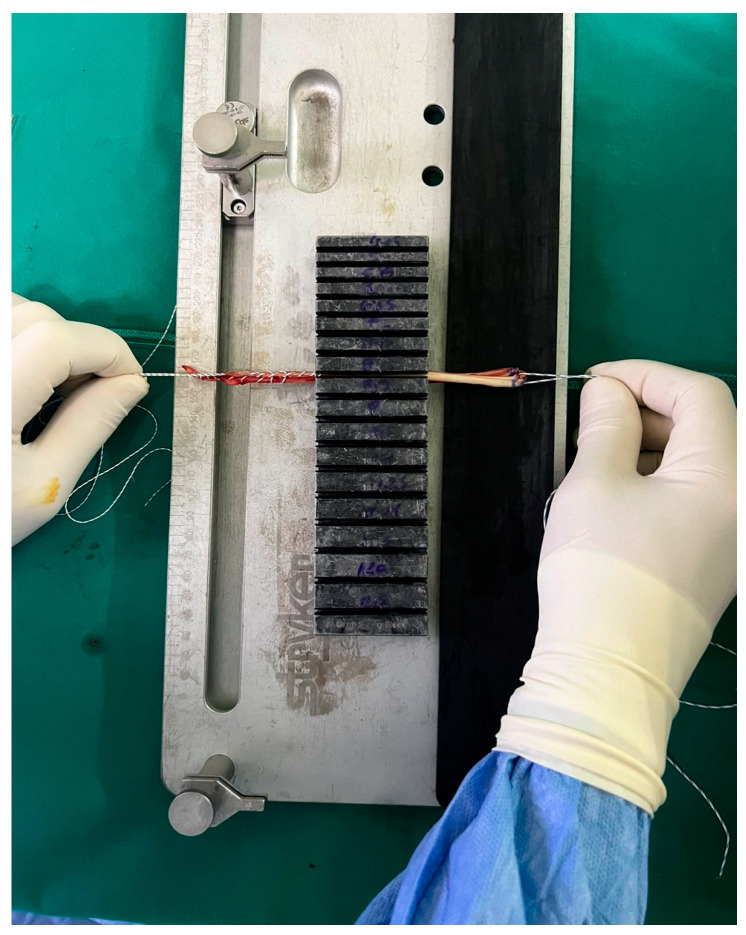
Diameter measurement of the four-strand hamstring graft.

All procedures were performed by the same fellowship-trained surgeon at the earliest opportunity after radiological and clinical diagnosis. A standard preoperative rehabilitation program was scheduled for all the patients. The same standard aseptic protocols and hamstring tendon harvesting technique were followed in all the patients. Firstly, both gracilis and semitendinosus tendons were harvested and prepared in a standardized fashion, as seen in Figure 1. Muscle tissue was removed from the tendons, and the length of each tendon was measured with a ruler and recorded in cm, rounded off to the nearest 0.5 cm. The diameter of the four-strand hamstring graft was measured using a graft sizer (Stryker Graft Measurement guide) calibrated to 0.5 mm (Figure 2). The diameter was defined as the smallest tunnel through which the graft could pass completely. The next step included arthroscopic knee exploration, confirmation of ACL rupture, and the assessment and repair of meniscal and cartilage lesions. Following bone tunnel preparation, the graft was fixed, and a final arthroscopy confirmed the success of this procedure and the absence of notch impingement.

### 2.3. Demographic Data and Anthropometric Measures

Preoperatively, data related to the age, gender, height, weight, and BMI of the patients were recorded. Thigh length and circumference measurements were obtained from the non-injured limb to avoid erratic measurements due to quadricep muscle atrophy, with the patients in a supine position and fully extended knees. The thigh length was measured from the anterior superior iliac spine to the superolateral pole of the patella. The thigh circumference was measured 15 cm proximally from the superolateral pole of the patella. All measurements were conducted by two members of the surgical team, and a final additional measurement was made by the leading surgeon when no agreement could be achieved.

### 2.4. Statistical Analysis

A mixed set of data with quantitative and qualitative variables were available. Two of the quantitative variables, graft diameter and length, were defined to be the dependent variables in this study. All quantitative variables were described with mean ± SD (standard deviation) and correlated in a pairwise manner with Pearson’s r correlation. The qualitative variables were described with the absolute count (relative % percent) per categorical level, and their dependency on any quantitative (dependent) variable was assessed via Student’s *t*-test. All visualizations were used as scatterplots for illustrating linear correlations, accompanied by linear trendlines. All statistical tests were considered two-sided, and statistical significance was taken when *p* < 0.05. The R language for statistical computing (version 4.3.2 (31 October 2023—“Eye Holes”) and RStudio IDE (version 2023.12.0+369 (10 January 2024)—“Ocean Storm”) were utilized in order to perform the overall statistical data analysis, processing, and visualization of the final results.

## 3. Results

This study initially recruited fifty consecutive patients, but one of them was excluded because of the use of an allograft. The final cohort comprised 49 patients, with an average ± SD age of 28.2 ± 9.3 years, with 39 (79.6%) of them being male and 10 of them female (20.4%). The average ± SD body mass index (BMI) value of the patients was equal to 26.6 ± 4.4 kg/m^2^, while 28 (57.2%) had injuries on the right side.

### 3.1. Correlation of Variables with Graft Diameter

**Figure 3:**
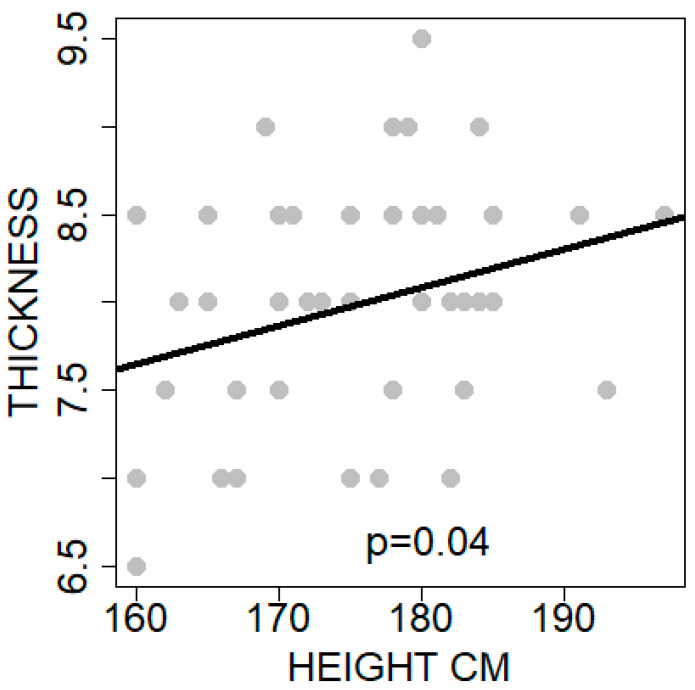
Diagram of the correlation between four-strand graft diameter and the height of the patients. The trendline shows positive correlation.

The average ± SD four-strand hamstring graft diameter was equal to 8 ± 0.7 mm. Table 1 illustrates the descriptive statistics of the variables contained in the dataset. Evaluating the correlations between each predictor individually and the diameter variable, only a single predictor was found to be statistically significantly correlated. This predictor was the patients’ height, with a medium correlation of Pearson r equal to 29.5%, *p* = 0.040 (Figure 3). The rest of the quantitative predictors, including gender, were not found to be statistically significant.

### 3.2. Correlation of Variables with Graft Length

**Figure 4:**
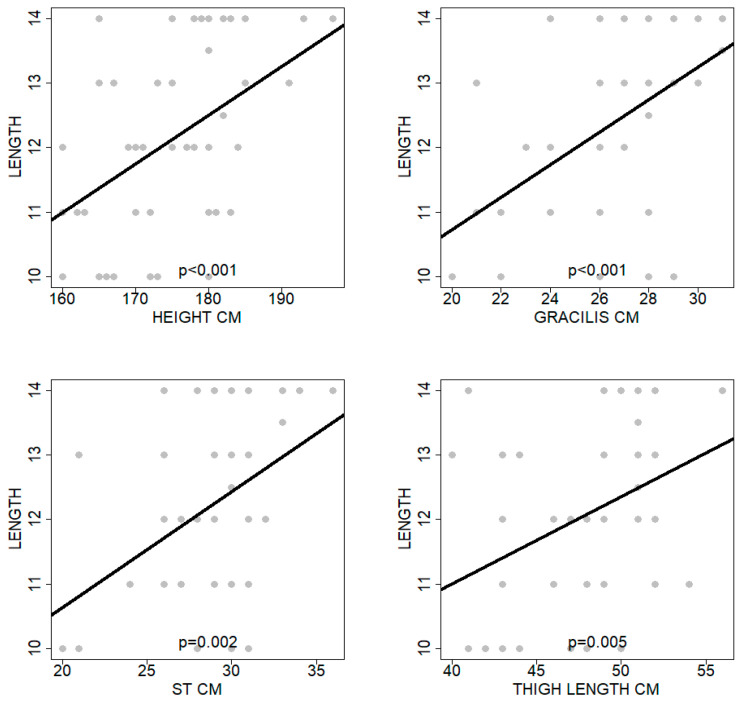
Diagrams depicting the correlations among four-strand hamstring graft length and patients’ height, the length of the gracilis and the semitendinosus tendons, and the thigh length. A trendline in all four subfigures shows positive correlation in any of the outcomes.

Assessing the dependencies between each predictor individually and the length variable, several of the assessed predictors were found to affect the outcome in a statistically significant way. Among these parameters, the length of the gracilis was found to be related to the strongest correlation of Pearson r equal to 57.58%, *p* < 0.001, while the patients’ height was found to be related to a strong correlation of Pearson r equal to 48.29%, *p* < 0.001. Furthermore, the length of the semitendinosus tendon showed a medium to strong correlation of Pearson r equal to 43.60%, *p* = 0.002, and the thigh length a medium correlation of Pearson r equal to 39.17%, *p* = 0.005 (Figure 4). Table 2 includes an in-depth presentation of the aforementioned inferential outcomes of the overall analysis.

## 4. Discussion

One of the drawbacks of the hamstring autografts used for ACL reconstruction is the variability in graft size, which is revealed to the surgeon only once the graft has already been harvested. Traditionally, a minimum hamstring tendon length of 28 cm, with a minimum diameter of 7 mm, was thought to be adequate for this type of autograft [15]. However, recent studies correlate a failure trend with a graft diameter smaller than 8 mm, while grafts larger than 10 mm fail at the fixation screws, as a result of traction [30]. The accurate assessment of patients undergoing ACL reconstruction in danger for insufficiently sized grafts could aid better preoperative planning. Thus, the main finding of this study was that anthropometric data can provide valuable preoperative information for quadruple hamstrings graft sizes in ACL reconstruction. The patients’ height and thigh length, along with the length of the gracilis and the semitendinosus, were positively correlated with the length of the four-strand hamstring graft. This is in accordance with the published literature, which reveals that researchers have already tried to correlate anthropometric data with graft size to predict insufficiency. Previous studies with a larger population reported strong correlations between a patient’s height and graft thickness [12,16,17,26,31,32]. Ho et al. reported a particularly strong correlation in female patients between the height, weight, and hamstring graft diameter [19]. Moghamis et al., in 2020, reported a significant positive moderate correlation between patients’ height and graft length, but only age had demonstrated a correlation with the final graft diameter [33]. The age, gender, and thigh length and circumference had no statistically significant correlation with the graft diameter in the present study. Treme et al. reported thigh circumference as the best predictor in men and the only important predictor in women in a study with a similar population [13]. Asif et al., studying an equivalent sample size, reported thigh circumference and height to be significant predictors of graft diameter [34]. Even though most studies agree that height is a key factor for the final graft’s quality, reports on age, gender, and BMI are still contradictory in the current literature [12,28,33,35]. According to Tuman et al., women tend to present hamstring tendon grafts with significantly smaller diameters, and grafts from patients with a height less than 149 cm are more likely insufficient (diameter smaller than 7 mm) [12]. In the present study, a young cohort of patients with a mean age of 28.2 ± 9.3 years was analyzed. The mean BMI was 26.6 ± 4.4 Kg/m^2^ with the majority of patients being male. There was no statistically significant correlation between the four-strand graft and these variables. Jansen et al., in a similar 2015 study of a Caucasian population, reported a correlation between gender and graft diameter <8 mm, stating that women more frequently had a final graft <8 mm compared to men. Other studies reported that age and gender could not be predictors of graft diameter but a BMI over 25 kg/m^2^ could predict a graft diameter >7 mm [14,20]. In a recent study of 160 patients who underwent primary ACL reconstruction, Goyal et al. reported no statistical significant correlation of the BMI to the quadrupled hamstrings graft [18]. Variations in hamstring graft size in different ethnic groups have recently become popular in the literature. This study reports results from a White Caucasian population. Jansen et al., in 2015, also reported the positive relation between the gracilis and semitendinosus length and Caucasian patients’ height, studying a much larger group of patients. The authors found a correlation between gender and a graft thickness of more than 8 mm [26]. Chiang et al. compared a Chinese patient population with a Caucasian population and stated that significant longer hamstring tendons were found among the Caucasian group [22]. Anatomical differences in these ethnicities seem not clearly defined, even though there are studies supporting smaller ACL lengths and diameters among Chinese patients and the higher possibility for smaller hamstring tendons even among patients of the same height [13,36]. Chinese Han patients who underwent double-bundle ACL reconstruction were studied by Xie et al. The gracilis and semitendinosus were separately quadrupled and significantly correlated with weight, height, and BMI but not gender [23]. Another study using anthropometry in a South Indian patient population undergoing ACL reconstruction found no correlation of the final graft size with age, gender, weight, BMI, and level of activity. Height was the only variable that expressed a positive relation with graft length and diameter [25]. A most recent study among the Bengali population reported that BMI, body weight, or body height cannot be used for predicting semitendinosus and gracilis tendon graft diameter [27]. Similarly to that, two studies reported no correlation between graft diameter and height or any other aforementioned parameter in an Asian male patient population [24,37]. In a very recent study of a Nepalese patient population, thigh length and patient height were strongly correlated with the graft size. Gender and BMI were non-significant [38].

Our study is characterized by several limitations, including the small sample size of the population under investigation, totaling 49 patients (sufficient according to the power analysis conducted), and the uneven distribution of female and male patients. Even if other interesting parameters including previous physical activity levels were not studied, these could be incorporated in future study designs. However, among the strong points of this study is the fact that this one is only the second work reporting results from a White Caucasian population. Multi-center studies combining different ethnicities could be another promising area for future studies.

Different parameters can influence the graft size used for ACL reconstruction, resulting in undersized ones and possible failure. The outcomes of this study support the literature published so far and prove that specific anthropometric data can be of assistance to the surgeon’s preoperative plan regarding graft choice in ACL reconstruction. The design of a predictive model of graft size depending on specific anthropometric data, incorporating parameters from all the studies mentioned in this paper, could have future clinical use and provide better final outcomes.

## 5. Conclusions

According to the results of this study, patient height, thigh length, and the length of the gracilis and semitendinosus tendons are valuable predictors of hamstrings grafts’ quality. Thus, the evaluation of theses parameters could aid better preoperative planning and guide surgeons’ choice toward other graft options in cases of predicted insufficiency.

## Figures and Tables

**Table 1:** Descriptive demographic statistics of the available variables in the data, independent and dependent. The quantitative variables are represented as the mean ± SD, and the qualitative variables are represented as the count (%) of their categorical level.

Variable	Valid Count	Statistic
Side	49	
Left	21	(42.9%)
Right	28	(57.2%)
Gender	49	
Female	10	(20.4%)
Male	39	(79.6%)
Age (years)	49	28.2 ± 9.3
Height (cm, centimeters)	49	175.3 ± 8.8
Weight (kg, kilograms)	49	81.5 ± 15
Body mass index (BMI)	49	26.6 ±4.4
Gracilis (cm)	49	25.6 ± 3.2
Semitendinosus (cm)	49	28.4 ± 3.3
Thigh length (cm)	49	48.4 ± 4
Thigh circumference (cm)	49	53.1 ± 6.1
Diameter	49	8 ± 0.7
Length	49	12.2 ± 1.4

**Table 2:** Inferential analysis for associating each predictor separately with the thickness and the length. Except for sex, the only qualitative predictor (assessed by Student’s *t*-test), all the other predictors were evaluated with Pearson’s r linear correlation. Significance is considered if *p* < 0.05.

Variable	Statistic (Diameter)	*p*-Value (Diameter)	Statistic (Length)	*p*-Value (Length)
Gender		0.070		0.587
Female	7.6 ± 0.7		11.9 ± 1.6	
Male	8.1 ± 0.6		12.2 ± 1.3	
Age (years)	−26.3%	0.068	−1.56%	0.915
Height (cm, centimeters)	29.5%	0.040	48.3%	<0.001
Weight (kg, kilograms)	17.3%	0.235	23.2%	0.109
Body mass index (BMI)	8%	0.583	−2.3%	0.877
Gracilis (cm)	12.3%	0.375	57.6%	<0.001
Semitendinosus (cm)	14.5%	0.321	43.6%	0.002
Thigh length (cm)	25.9%	0.073	39.2%	0.005
Thigh circumference (cm)	25.6%	0.076	9.3%	0.528

## Data Availability

The data presented in this study are available upon request from the corresponding author. The data are not publicly available due to privacy restrictions.
